# Comparison of long-term prognoses between 3D-printed template-guided and conventional freehand interstitial brachytherapy for locally advanced cervical cancer: a single-center retrospective study

**DOI:** 10.3389/fonc.2026.1766456

**Published:** 2026-03-24

**Authors:** Jun-Jun Miao, Xu-Yu Zhang, Yong-Xia Zhang, Lei Gao, Lu-Jun Zhao

**Affiliations:** 1 Hebei Province Cangzhou Hospital of Integrated Traditional Chinese Medicine and Western Medicine, Cangzhou, China; 2 Hebei Province Integrated Traditional Chinese and Western Medicine 3D Printing Technology Innovation Center, Cangzhou, China; 3 Tianjin Medical University Cancer Institute & Hospital, Tianjin, China

**Keywords:** 3D-printed template, cervical cancer, dosimetry, interstitial brachytherapy, survival analysis

## Abstract

**Objective:**

Conventional freehand three-dimensional interstitial brachytherapy (F-3DIB) for locally advanced cervical cancer (LACC) is limited by suboptimal dose distribution and operator dependency. This study aimed to evaluate the long-term survival and safety benefits of 3D-printed template-guided interstitial brachytherapy (3D-PTIB).

**Methods:**

This retrospective single-center cohort study included 300 patients with FIGO IIB-IVA LACC who underwent brachytherapy between January 2021 and June 2022. The median follow-up time was 36.2 months (range: 34-41). Follow-up data were collected between January 2024 and June 2025, ensuring adequate follow-up for survival assessment. Patients were divided into 3D-PTIB and F-3DIB groups. Propensity score matching was used to balance baseline characteristics, resulting in a matched cohort of 240 patients. The outcomes assessed included 3-year overall survival (OS), progression-free survival (PFS), local control (LC), dosimetric parameters, and late toxicities.

**Results:**

The 3D-PTIB group exhibited significantly better 3-year OS (76.7% vs. 66.2%, HR = 0.69, p = 0.02) and LC (90.0% vs. 78.3%, HR = 0.47, p = 0.01) than the F-3DIB group. A trend toward improved 3-year PFS was also observed (70.8% vs. 62.3%, HR = 0.73, p = 0.05). Notably, the 3D-PTIB group achieved an 11.7% absolute improvement in local control (90.0% vs. 78.3%). A *post hoc* power analysis indicated that the study had approximately 80% power (α = 0.05, two-sided) to detect the observed hazard ratio of 0.69 for OS in the matched cohort. Dosimetric advantages included a significantly greater HR-CTV D90 (87.5 ± 3.2 Gy vs. 82.1 ± 4.8 Gy, p < 0.001) and reduced rectal D2 cc (64.5 ± 5.1 Gy vs. 72.3 ± 6.7 Gy, p < 0.001). Clinically, the incidence of ≥ grade 3 radiation proctitis was significantly lower in the 3D-PTIB group (4.2% vs. 11.7%, p = 0.008).

**Conclusion:**

3D-printed template-guided interstitial brachytherapy significantly improves intermediate- to long-term survival and reduces late toxicity in LACC patients, particularly high-risk patients with a tumor diameter > 4 cm or parametrial invasion, highlighting its potential as a superior alternative to conventional freehand interstitial brachytherapy.

## 1 Introduction

Cervical cancer remains a significant global health burden, ranking as the fourth most common malignancy and the fourth leading cause of cancer-related deaths among women worldwide (1, 2). In 2020, an estimated 604,000 new cases and 342,000 deaths were reported globally, with over 85% of these cases occurring in lowland middle-income countries (3). In China, cervical cancer is the second most prevalent gynecologic malignancy, with age-standardized incidence and mortality rates of 10.7 and 3.3 per 100,000 women, respectively (4). Notably, approximately 40–50% of patients present with locally advanced cervical cancer (LACC, FIGO stages IIB–IVA) at diagnosis, characterized by a large tumor diameter (> 4 cm), parametrial invasion, or vaginal extension (5, 6). These patients face a grim prognosis, with 3-year survival rates stagnating at 50–65%, despite advances in multimodal therapy (7).

Concurrent chemoradiation therapy (CCRT) has been the standard treatment for LACC for over two decades, with management typically involving a combination of CCRT and brachytherapy to optimize treatment outcomes (8). However, conventional intracavitary brachytherapy (ICBT) using tandem-and-ovoid applicators faces inherent limitations in treating bulky or asymmetrical tumors, often resulting in suboptimal dose coverage (< 80 Gy to HR-CTV D90) and excessive organ at risk (OAR) exposure (9). To address these challenges, interstitial brachytherapy (ISBT) has emerged as a critical modality for LACC, particularly in patients with parametrial involvement, vaginal stenosis, or tumors extending beyond the cervical os (10). An early study demonstrated that combining ICBT with ISBT increased the HR-CTV D90 by 15–20% compared with that of ICBT alone, resulting in a 10–15% improvement in local control (11). Nevertheless, conventional freehand 3D interstitial brachytherapy (F-3DIB) relies heavily on operator expertise, leading to inconsistent needle placement, prolonged procedure times, and variable dosimetric outcomes (12). A multicenter analysis by Fokdal et al. (13) revealed that only 62% of conventional freehand interstitial brachytherapy plans met the GEC-ESTRO dose constraints for OARs, with the rectal D2 cc exceeding 75 Gy in 28% of cases. These shortcomings underscore the urgent need for standardized, operator-independent techniques.

3D-printed template-guided interstitial brachytherapy (3D-PTIB) utilizes patient-specific applicators designed from preoperative imaging to preset needle trajectories, ensuring anatomical conformity and dosimetric reproducibility (14). Compared with traditional templates, 3D-printed personalized templates offer significant advantages. These templates can be predesigned and fabricated, enabling the optimization of key parameters such as the number, angle, and insertion depth of noncoplanar needles (15). This approach simplifies the procedure, facilitates needle adjustments, and improves needle fixation, ultimately resulting in a more effective therapeutic dose distribution. Logar et al. (16) highlighted the utility of a 3D-printed personalized applicator for image-guided adaptive intracavitary/interstitial brachytherapy, particularly for patients with stage IIIB and IVA cervical cancer, where standard applicators often fail to achieve ideal dose coverage. Their findings demonstrated an increase of 8–9 Gy in high-risk clinical target volume (HR-CTV) prescriptions, underscoring the potential of 3D-printed templates to increase treatment efficacy.

3D-printed template-guided interstitial brachytherapy utilizes a patient specific, digitally designed template, departing from the standardized template of conventional brachytherapy. This enables an optimized, individualized needle arrangement that improves target coverage, organ sparing, dosimetry, and procedural reproducibility. This single-center retrospective study aimed to address these gaps by analyzing one of the LACC cohorts treated with 3D-printed template-guided interstitial brachytherapy. Compared with conventional freehand interstitial brachytherapy, 3D-printed template-guided interstitial brachytherapy is hypothesized to improve 3-year overall survival (OS) and local control (LC) through enhanced dose escalation, reduce ≥ Grade 3 late toxicities by minimizing exposure to OARs, and stratify clinical benefits on the basis of tumor diameter and parametrial invasion status. By integrating dosimetric, survival, and toxicity endpoints across diverse clinical settings, this study provides actionable insights to optimize brachytherapy in the era of precision oncology.

## 2 Materials and methods

### 2.1 Study design and ethical considerations

This single-center retrospective study enrolled patients who underwent brachytherapy procedures during the treatment period from January 2021 to June 2022. To assess intermediate- to long-term outcomes, we conducted dedicated follow-up data collection and analysis for this study between January 2024 and June 2025. This period was dedicated to ascertaining and determining the final status (e.g., survival, recurrence) of all enrolled patients for analysis. This approach ensured a minimum potential follow-up of approximately 24 months, allowing for robust assessment of 3-year survival endpoints. The median follow-up duration for the matched cohort was 36.2 months (range: 34-41). The study protocol was approved by the Ethics Committee of Hebei Province Cangzhou Hospital of Integrated Traditional and Western Medicine. In routine clinical practice during the study period, the choice between 3D-printed template-guided and conventional freehand interstitial brachytherapy was primarily guided by tumor characteristics and anatomical complexity rather than patient age or performance status. 3D-printed template-guided interstitial brachytherapy was systematically recommended for patients with at least one of the following criteria: tumor diameter > 4 cm, parametrial invasion on prebrachytherapy MRI, or inadequate HR-CTV coverage (D90 < 85 Gy) on virtual planning using standard intracavitary applicators. Conventional freehand interstitial brachytherapy was used for patients with more favorable anatomy or when 3D-printed template-guided interstitial brachytherapy was logistically unavailable (e.g., during the initial implementation phase). This selection protocol aimed to prioritize 3D-printed template-guided interstitial brachytherapy for cases expected to benefit most from personalized needle guidance while minimizing allocation bias based on nonclinical factors.

### 2.2 Data sources and patient selection

A total of 872 patients diagnosed with FIGO IIB-IVA cervical cancer were initially screened from institutional databases. The inclusion criteria were as follows: (1) histologically confirmed squamous cell carcinoma or adenocarcinoma of the cervix; (2) FIGO 2018 stage IIB-IVA disease, confirmed by pelvic MRI and PET-CT; (3) completion of definitive treatment, CCRT with external beam radiotherapy (EBRT) followed by ISBT; and (4) having follow-up data available for collection and assessment during the dedicated study follow-up period (January 2024 to June 2025). The exclusion criteria were as follows: (1) distant metastasis (FIGO IVB) at diagnosis; (2) nonepithelial histology (e.g., small cell carcinoma, sarcoma); (3) prior pelvic radiotherapy or radical surgery; and (4) incomplete medical records or imaging data.

**Figure 1:**
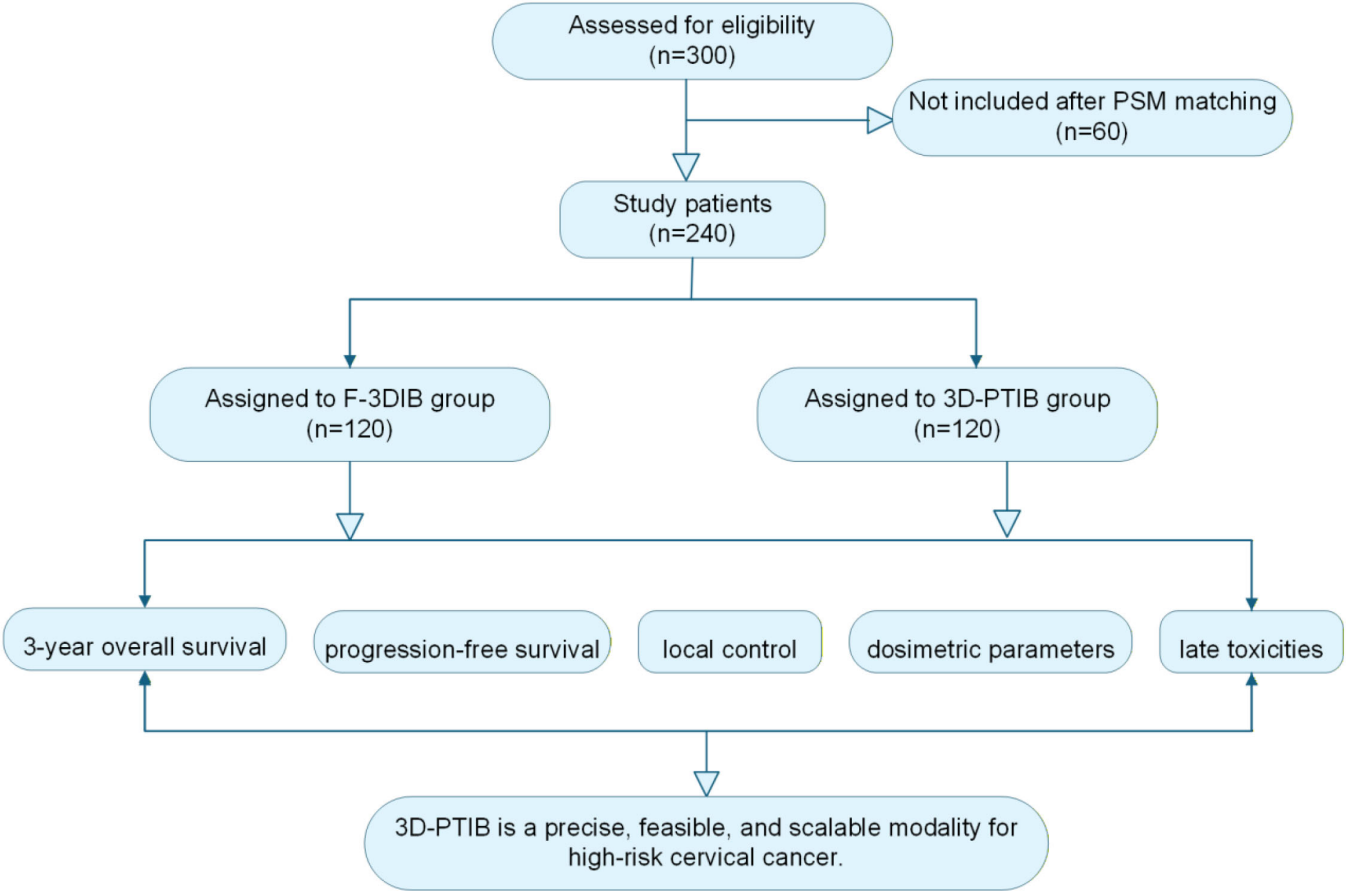
Flowchart of the study selection process.

Among the 872 initially screened patients, 572 were excluded for the following reasons: distant metastasis at diagnosis (n = 89), non−epithelial histology (e.g., small cell carcinoma, sarcoma; n = 47), prior pelvic radiotherapy or radical surgery (n = 132), incomplete medical records or imaging data (n = 304). A total of 300 patients met the eligibility criteria and were stratified into two groups: the 3D-PTIB group (n = 152), consisting of patients treated with 3D-printed template-guided ISBT, and the F-3DIB group (n = 148), consisting of patients treated with freehand ISBT (**Figure 1**).

### 2.3 Treatment protocols

All patients received intensity-modulated radiotherapy (IMRT) via a 6 MV linear accelerator. The clinical target volume (CTV) included the primary tumor, entire uterus, parametria, upper 1/3 of the vagina, and regional lymph nodes. The planning target volume was generated with a 7–10 mm margin around the CTV. A total dose of 45–50.4 Gy was delivered in 25–28 fractions (1.8–2.0 Gy/fraction), five fractions per week. Cisplatin (40 mg/m² weekly) was administered during EBRT unless contraindicated. Dose modifications followed the National Comprehensive Cancer Network (NCCN) guidelines for hematologic/nonhaematologic toxicity.

**Figure 2:**
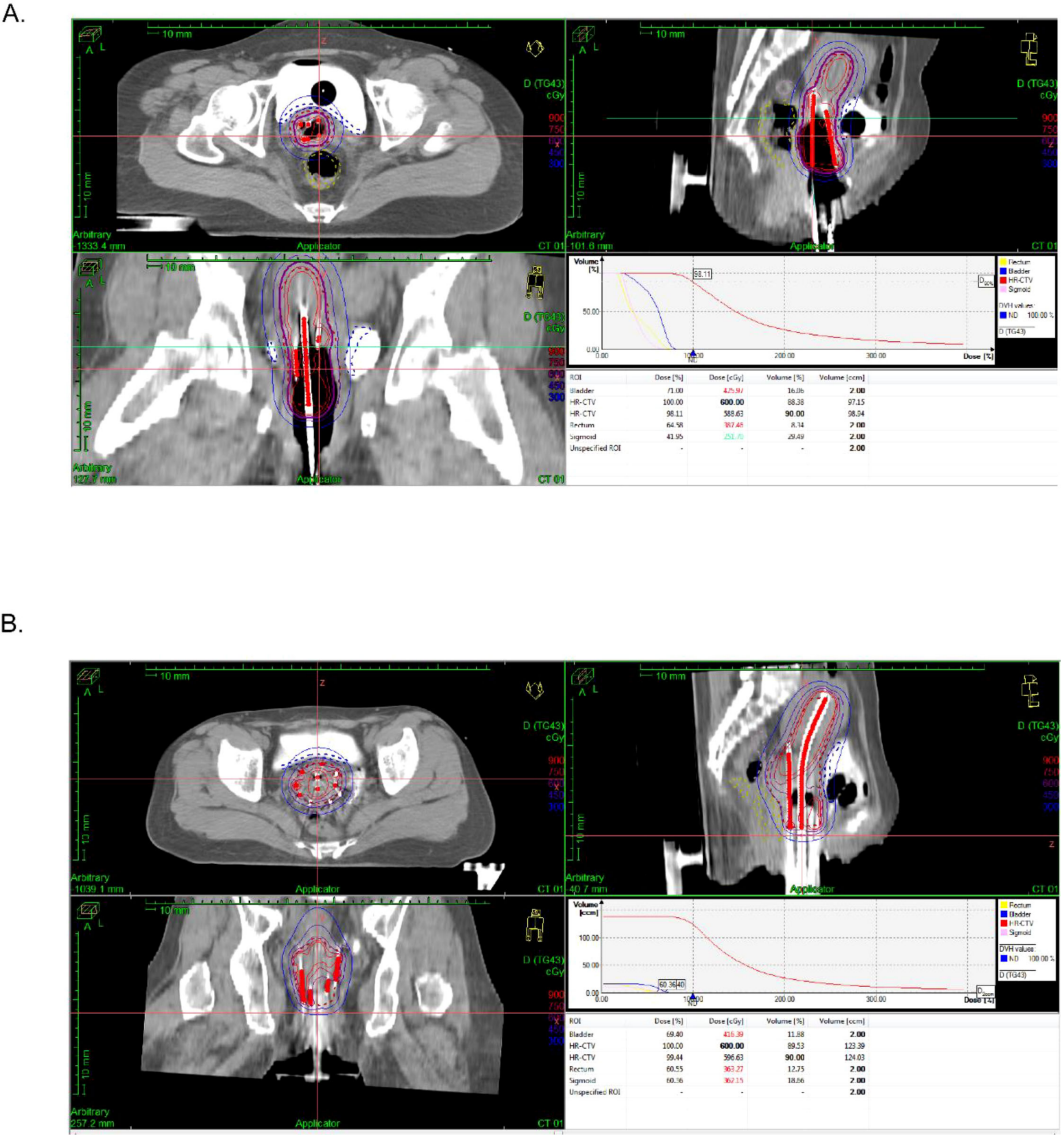
Treatment implementation protocols for F-3DIB and 3D-PTIB. **(A)** Treatment protocol for F-3DIB. **(B)** Treatment protocol for 3D-PTIB.

Conventional freehand interstitial brachytherapy (F-3DIB): (1) Needle insertion: Under transrectal ultrasound guidance, 6–12 interstitial needles were manually inserted through a standard commercial perineal template (Varian Gyne^®^). Intraoperative CT confirmed the position of the needle. (2) Dose constraints mirrored the 3D-printed template-guided interstitial brachytherapy protocol (**Figure 2A**). The OAR dose constraints (rectum D2 cc ≤ 75 Gy, bladder D2 cc ≤ 90 Gy, and sigmoid D2 cc ≤ 75 Gy) adhered to the same GEC-ESTRO guidelines as referenced for the 3D-PTIB group.

3D-printed template-guided interstitial brachytherapy: (1) Simulation Imaging: High-resolution CT and T2-weighted MRI were performed 24–48 hours prior to brachytherapy. Patients were immobilized in the lithotomy position with a rectal balloon (inflated with 60 mL of air) and vaginal gauze packing for organ stabilization and displacement. (2) Target and Organ-at-Risk Delineation: Contouring followed GEC-ESTRO guidelines. The HR-CTV was defined according to GEC-ESTRO recommendations, based on the residual gross tumor volume (GTV) identified on T2-weighted MRI at the time of brachytherapy, incorporating the tumor extension at diagnosis, the pattern of tumor regression after EBRT, and clinical examination findings. A 5-mm isotropic margin was added to this GTV to encompass potential microscopic disease while respecting anatomical barriers such as the bladder, rectum, and pelvic sidewall. The IR-CTV included the HR-CTV with an additional 10-mm margin, respecting anatomical barriers. (3) Patient-Specific 3D Template Design and Fabrication: a. Data Acquisition and Fusion: High-resolution simulation CT and T2-weighted MRI were imported into a dedicated brachytherapy treatment planning system (Oncentra Brachy, Elekta). The images were fused using bony anatomy and soft-tissue landmarks. b. Virtual Planning and Needle Path Design: Contours of HR-CTV, IR-CTV, and OARs (bladder, rectum, sigmoid, ureters) were delineated on the fused images following GEC-ESTRO guidelines. Critically, needle trajectories were planned based solely on optimal coverage of the HR-CTV and avoidance of OARs and bony structures (e.g., pubic arch, sacrum) without being constrained by the fixed hole pattern of a standard template. The TPS’s virtual needle module was used to simulate trajectories, ensuring a minimum distance of 3–5 mm from critical structures. Noncoplanar angles were frequently employed to encompass lateral parametrial disease. Typically, 8 to 14 needles (median: 11) were planned and used, which was comparable to the range used in the F-3DIB group (6–12 needles). c. Digital template modeling: The optimized needle entry points and angles on the perineal surface were exported. Using 3D modeling software (3-matic), a digital template model was created. d. 3D Printing and Postprocessing: The final digital model (in STL format) was processed with slicing software and fabricated using a biocompatible, medical-grade photopolymer resin (e.g., Dental SG Resin, Formlabs) on a high-resolution stereolithography (SLA) 3D printer (Form 3B+, Formlabs). After printing, the template was washed in isopropanol to remove uncured resin and postcured under UV light to achieve maximum mechanical strength and biocompatibility. e. Quality Assurance and Sterilization: The physical template was visually inspected, and its key dimensions were verified against the digital model. Channels were checked for patency. Prior to each procedure, the template was sterilized using low-temperature hydrogen peroxide plasma (STERRAD) according to institutional protocols. (4) Strategies for Specific Anatomical Challenges: a. Sigmoid Colon Protection: To avoid inadvertent penetration, the sigmoid’s course was evaluated on preprocedural imaging. Trajectories within 5 mm were modified. Vaginal packing and rectal balloons were used for displacement, and thinner needles were selected when feasible. Final needle positions were verified intraoperatively with imaging fusion. b. Bladder Proximity or Invasion: For anterior disease near or involving the bladder (HR-CTV–bladder wall distance ≤ 5 mm on MRI), needle paths were oriented laterally. When transvesical needles were unavoidable, they were planned for perpendicular entry at the bladder base with minimal intravesical length, guided by real-time ultrasound. An additional bladder wall dose constraint (D0.1 cc < 85 Gy) was applied alongside the standard D2 cc limit. A Foley catheter was maintained for 24–48 hours post-procedure if transvesical insertion was performed. c. Ureteral Protection: Ureters were delineated on T2-MRI and expanded by 5 mm to create a Planning Risk Volume (PRV). Needle trajectories were adjusted to avoid the PRV. A dose constraint of D2 cc < 75 Gy EQD2 (α/β = 3) was applied. d. Rectal Proximity: For posterior tumors abutting the rectum (HR-CTV ≤ 5 mm), posterior needles were angled laterally through the pararectal space. Dwell positions were shifted anteriorly, and a stricter rectal wall constraint (D0.1 cc ≤ 70 Gy) was used. Needle tips were verified to be ≥ 3 mm from the mucosa. e. Tissue Fibrosis and Needle Deflection: To counteract post-EBRT fibrosis, rigid, stylet-equipped needles were inserted using a rotational technique. Intraoperative cone-beam CT (CBCT) fusion verified alignment with planned paths. Deviations > 2 mm prompted reinsertion or replanning. f. Vaginal Stenosis: Mild to moderate stenosis was managed with intraoperative dilation. Severe stenosis or deformity warranted a hybrid approach using a customized proximal template combined with perineal interstitial needles. (5) Intraoperative Execution and Validation: The sterilized template was fixed in position. Needles were inserted, and their placement accuracy was immediately verified using intraoperative C-arm fluoroscopy or CBCT. Any deviation > 2 mm triggered reassessment and corrective replanning. Following insertion, a customized thermoplastic mesh was applied for external compression and stability. A pre−fraction CBCT confirmed overall alignment. (6) Dose Prescription and Delivery: All efforts were directed toward achieving an HR-CTV D90 ≥ 85 Gy while strictly adhering to OAR dose constraints: rectum D2 cc ≤ 75 Gy, bladder D2 cc ≤ 90 Gy, and sigmoid colon D2 cc ≤ 75 Gy. (7) Contingency Planning for Unsuitable Cases: If virtual planning, after exhaustive trajectory optimization, could not achieve both HR−CTV D90 ≥ 85 Gy and OAR constraints, patients were not considered candidates for standard 3D-printed template-guided interstitial brachytherapy. For such cases, two alternatives were enacted: a. Hybrid Intracavitary/Interstitial Approach: A standard applicator combined with limited, ultrasound-guided freehand interstitial needles was used. b. EBRT Boost: If the hybrid approach remained insufficient, a conformal EBRT boost (5–10 Gy in 2–5 fractions) was delivered to the undercovered volume identified on fused CT-MRI, aiming to achieve a cumulative EQD2 approaching the target while respecting OAR limits (**Figure 2B**).

It is important to note a methodological difference in imaging for treatment planning between the two groups. The 3D-printed template-guided interstitial brachytherapy protocol utilized preplanning with high-resolution CT and T2-weighted MRI, while the conventional freehand interstitial brachytherapy procedure relied on intraoperative CT for final planning and needle verification. Magnetic resonance imaging (MRI) offers superior soft-tissue contrast for delineating the gross tumor volume and parametrial extension compared to CT. Consequently, the HR-CTV volumes defined in the 3D-PTIB group, based on fused CT/MRI, might be more comprehensive, particularly for parametrial disease, than those defined using CT alone in the F-3DIB group. This systematic difference in imaging modality could influence the absolute values of dosimetric parameters (e.g., HR-CTV volume, D90) when comparing the two techniques. The clinical and survival outcomes, however, remain the primary endpoints of this comparison.

### 2.4 Clinical outcomes

The survival endpoints included 3-year OS, defined as the time from brachytherapy completion to death from any cause; PFS, defined as the time to local or distant recurrence or death; and LC, defined as the absence of pelvic recurrence confirmed by MRI or PET-CT. Toxicity assessment focused on late toxicities (≥ 3 months post-treatment), graded according to CTCAE v5.0, with categories for gastrointestinal (radiation proctitis, fistula, stenosis), genitourinary (cystitis, ureteral stricture, vesicovaginal fistula), and vaginal (necrosis, stenosis) toxicities.

### 2.5 Statistical analysis

Statistical analysis was performed using R software (version 4.3.2; R Foundation for Statistical Computing, Vienna, Austria). Missing data were addressed primarily through complete-case analysis, as patients with incomplete medical records or imaging data were excluded during the initial screening process. To assess the potential impact of missing data on the primary survival outcome, a sensitivity analysis using multiple imputation by chained equations (MICE) for key covariates (age, tumor diameter) was performed and compared with the complete-case analysis; the results were consistent. To reduce selection bias, PSM was performed. The matching variables included age, FIGO stage, tumor diameter, and number of chemotherapy cycles. A 1:1 nearest-neighbor matching algorithm with a caliper of 0.1 standard deviation was applied, and balance was evaluated via standardized mean differences (SMDs <0.1) and variance ratios (0.8–1.2). Survival analysis was performed via Kaplan–Meier estimates for OS, PFS, and LC, with comparisons conducted via log-rank tests. Hazard ratios (HRs) with 95% confidence intervals (CIs) were derived from univariable and multivariable Cox proportional hazards regression models. The proportional hazards assumption was tested and confirmed using Schoenfeld residuals (all p > 0.05). Dosimetric and toxicity analyses compared continuous variables via Student’s t test or the Mann–Whitney U test and categorical variables via the chi-square test or Fisher’s exact test. All p values are two-sided, and statistical significance was set at p < 0.05. Confidence intervals are reported at the 95% level. Dose–response modeling utilizes restricted cubic splines (RCSs) with four knots to examine the nonlinear relationships between D90 and survival. To address the adequacy of the sample size in the matched cohort for the primary survival endpoint, a *post hoc* power calculation was performed using the observed hazard ratio (HR = 0.69), significance level (α = 0.05, two-sided), and the number of events (68 deaths) in the matched cohort (n = 240). This analysis indicated that the study had approximately 80% statistical power to detect the observed treatment effect for overall survival. Additionally, the minimum detectable effect size with 80% power given the current sample and event rate was approximately 0.67. Subgroup and sensitivity analyses were conducted to validate the findings, including complete-case analysis versus multiple imputation for missing data and inverse probability weighting, to confirm the robustness of the PSM results. Effect modification by tumor size (> 4 cm vs. ≤ 4 cm) and parametrial invasion status was formally assessed by including interaction terms in the Cox proportional hazards models, with significance evaluated using Wald tests. PSM was performed using a 1:1 nearest-neighbor algorithm with a caliper width of 0.1 standard deviation of the logit of the propensity score. The propensity score was estimated via logistic regression incorporating the following covariates: age, FIGO stage (IIB, III, IVA), tumor diameter, and number of chemotherapy cycles. Balance between the matched groups was assessed using standardized mean differences (SMDs), with an SMD <0.1 considered indicative of good balance, and variance ratios (target range: 0.8–1.2). To explore the potential nonlinear relationship between the total EQD2 of HR-CTV D90 and survival outcomes, RCS with four knots (placed at the 5th, 35th, 65th, and 95th percentiles of the D90 distribution) were incorporated into Cox proportional hazards models. The nonlinearity was tested using the Wald test for the combined spline terms. Multivariable Cox models identified independent predictors of 3-year OS, with treatment modality (3D-PTIB vs. F-3DIB) as the primary exposure. A separate model assessed the continuous variable HR-CTV D90 (Gy). Models were adjusted for age, FIGO stage, tumor diameter, parametrial and nodal involvement, and chemotherapy completion. Proportional hazards were verified using Schoenfeld residuals. Restricted cubic spline analysis, similarly adjusted, evaluated the independent dose–response relationship between D90 and survival risk. To further validate the robustness of our findings, we conducted a sensitivity analysis using inverse probability weighting (IPW). Interaction tests for effect modification by tumor size (> 4 cm vs. ≤ 4 cm) and parametrial invasion status were prespecified. The propensity score model included all matching variables, and weights were calculated using the standardized mortality ratio weighting method. Weighted Cox models were fitted for OS, PFS, and LC, and the results were compared with the primary PSM analysis to assess consistency.

## 3 Results

### 3.1 Patient characteristics and matching outcomes

A total of 300 patients with LACC were initially enrolled. After applying the inclusion/exclusion criteria and PSM, 240 patients were included in the final analysis. Prior to matching, SMDs for key covariates indicated some imbalance, particularly in tumor diameter (SMD = 0.15) and FIGO stage distribution (SMD for stage III = 0.12). PSM effectively balanced these characteristics, reducing all SMDs to below 0.05 (**Table 1**). The PSM process excluded 60 patients (3D-PTIB: n = 32; F-3DIB: n = 28) who lacked suitable matches within the specified caliper. The baseline characteristics of these excluded patients were not significantly different from those of the overall unmatched cohort. The matched cohort was used for all subsequent analyses to minimize confounding. After matching, patients were included in the final analysis, with baseline characteristics well balanced between the 3D-PTIB and F-3DIB groups. Post-PSM, the groups had comparable demographics, with a mean age of 51.7 ± 8.2 years in the 3D-PTIB group versus 52.1 ± 8.0 years in the F-3DIB group, and similar tumor diameters (4.6 ± 1.2 cm vs. 4.7 ± 1.3 cm). The FIGO stage distributions were also balanced, with 25.0% versus 23.3% for stage IIB, 65.0% versus 66.7% for stage III, and 10.0% versus 10.0% for stage IVA in the 3D-PTIB and F-3DIB groups, respectively. HPV-positive rates (78.3% vs. 77.5%), parametrial invasion (58.3% vs. 56.7%), lymph node involvement (41.7% vs. 40.8%), and concurrent chemotherapy utilization (93.3% vs. 94.2%) were closely aligned, with an SMD <0.1 for all variables. The median follow-up time for the matched cohort was 36.2 months (range: 34–41 months). During follow-up, 68 deaths (28.3%) and 52 recurrence events (21.7%) were recorded. To assess potential temporal biases, we examined the distribution of procedures over the study period. The proportion of 3D-printed template-guided interstitial brachytherapy cases remained stable throughout the enrollment window (52% in 2021 vs. 50% in 2022, p = 0.72), suggesting no significant learning-curve effect or sequential adoption bias.

**Table 1:** Baseline characteristics before and after propensity score matching.

Variable	Before matching		After matching	
	3D-PTIB (n = 152)	F-3DIB (n = 148)	3D-PTIB (n = 120)	F-3DIB (n = 120)
Age (years)	52.3 ± 8.5	51.8 ± 7.9	51.7 ± 8.2	52.1 ± 8.0
FIGO stage				
IIB (%)	38 (25.0%)	35 (23.6%)	30 (25.0%)	28 (23.3%)
III (%)	98 (64.5%)	96 (64.9%)	78 (65.0%)	80 (66.7%)
IVA (%)	16 (10.5%)	17 (11.5%)	12 (10.0%)	12 (10.0%)
Tumor diameter (cm)	4.8 ± 1.3	4.9 ± 1.4	4.6 ± 1.2	4.7 ± 1.3
HPV-Positive	118 (77.6%)	112 (75.7%)	94 (78.3%)	93 (77.5%)
Parametrial Invasion	89 (58.6%)	85 (57.4%)	70 (58.3%)	68 (56.7%)
Lymph Node Involvement	63 (41.4%)	60 (40.5%)	50 (41.7%)	49 (40.8%)
Concurrent Chemotherapy	142 (93.4%)	138 (93.2%)	112 (93.3%)	113 (94.2%)

Standardized mean differences (SMDs) before matching: Age (0.06), FIGO stage IIB (0.03), III (0.12), IVA (0.03), tumor diameter (0.15), HPV-positive status (0.04), parametrial invasion (0.02), lymph node involvement (0.02), concurrent chemotherapy (0.01). After matching, all SMDs were <0.05, indicating excellent balance. Data are presented as the means ± SDs or n (%).

### 3.2 Superior dosimetry and reduced toxicity with 3D-PTIB

**Table 2** highlights the superior outcomes of the 3D-PTIB group compared with those of the F-3DIB group in terms of both target coverage and OAR sparing. For target coverage, the mean HR-CTV D90 was 5.4 Gy higher (87.5 ± 3.2 Gy vs. 82.1 ± 4.8 Gy; p < 0.001), and the HR-CTV V100 increased by 5.5% (98.2 ± 1.5% vs. 92.7 ± 3.1%; p < 0.001). Significant reductions in OAR doses were also observed: the rectum D2 cc decreased by 7.8 Gy (64.5 ± 5.1 Gy vs. 72.3 ± 6.7 Gy; p < 0.001), the bladder D2 cc decreased by 7.4 Gy (78.2 ± 4.3 Gy vs. 85.6 ± 5.9 Gy; p < 0.001), and the sigmoid D2 cc decreased by 6.7 Gy (62.8 ± 4.9 Gy vs. 69.5 ± 6.2 Gy; p < 0.001). Additionally, the conformity index (CI) was significantly better for 3D-PTIB (0.89 ± 0.04 vs. 0.76 ± 0.07; p < 0.001). 3D-PTIB resulted in a significant reduction in the incidence of ≥ Grade 3 radiation proctitis (5/120 [4.2%] vs. 14/120 [11.7%]; RR: 0.38; p = 0.008). Notably, no cases of vaginal necrosis occurred in the 3D-PTIB group, compared with 2/120 (1.7%) in the F-3DIB group.

**Table 2:** Dosimetric parameters and late toxicity outcomes: Comparison between the 3D-PTIB and F-3DIB groups.

Parameter	3D-PTIB (n = 120)	F-3DIB (n = 120)	Difference (95% CI)	*p*
Target coverage				
HR-CTV D90 (Gy)	87.5 ± 3.2	82.1 ± 4.8	5.4 (4.6–6.2)	<0.001
HR-CTV V100 (%)	98.2 ± 1.5	92.7 ± 3.1	5.5 (4.8–6.2)	<0.001
OAR doses				
Rectum D2 cc (Gy)	64.5 ± 5.1	72.3 ± 6.7	-7.8 (-9.1–6.5)	<0.001
Bladder D2 cc (Gy)	78.2 ± 4.3	85.6 ± 5.9	-7.4 (-8.6–6.2)	<0.001
Sigmoid D2 cc (Gy)	62.8 ± 4.9	69.5 ± 6.2	-6.7 (-7.9–5.5)	<0.001
Conformity Index (CI)	0.89 ± 0.04	0.76 ± 0.07	0.13 (0.11–0.15)	<0.001
Gastrointestinal				
Radiation Proctitis (≥ G3)	5 (4.2%)	14 (11.7%)	0.38 (0.14–0.98)	0.008
Rectal Fistula	1 (0.8%)	4 (3.3%)	0.25 (0.03–2.16)	0.18
Genitourinary				
Cystitis (≥ G3)	3 (2.5%)	7 (5.8%)	0.43 (0.11–1.63)	0.20
Vesicovaginal Fistula	1 (0.8%)	4 (3.3%)	0.25 (0.03–2.16)	0.18
Vaginal				
Stenosis (≥ G3)	4 (3.3%)	9 (7.5%)	0.44 (0.14–1.40)	0.16
Necrosis	0 (0%)	2 (1.7%)	–	0.50

Toxicity data are reported as the number of patients (percentage) and risk ratios (RRs) with 95% confidence intervals.

### 3.3 Impact of tumor diameter and parametrial invasion on 3D-PTIB efficacy in LACC​​

**Table 3** shows that for tumors > 4 cm, 3D-PTIB significantly improved 3-year OS by 13.3% (73.6% vs. 60.3%; HR = 0.61, p = 0.005) and demonstrated superior 3-year local control (LC: 85.4% vs. 71.2%; HR = 0.44, p = 0.003). In this bulky tumor subgroup, the incidence of ≥ Grade 3 radiation proctitis was also significantly lower with 3D-PTIB (5.6% vs. 16.4%, p = 0.03). In the tumor ≤ 4 cm subgroup (3D-PTIB: n = 48; F-3DIB: n = 47), the number of death events was limited (3D-PTIB: 9/48; F-3DIB: 13/47), and no significant OS difference was observed (HR = 0.89, p = 0.42) or LC (91.7% vs. 87.2%; HR = 0.65, p = 0.32). The rates of ≥ Grade 3 proctitis were similarly low in both groups (≤ 4 cm: 2.1% vs. 6.4%, p = 0.36).

**Table 3:** Subgroup analysis of 3-year OS/LC by tumor diameter and parametrial invasion.

Subgroup	3D-PTIB (n = 120)	F-3DIB (n = 120)	3-Year OS		HR (95% CI)	*p*
			(3D-PTIB)	(F-3DIB)		
Tumor diameter ≤ 4 cm	48	47	81.3%	72.3%	0.89 (0.54–1.47)	0.42
Tumor diameter > 4 cm	72	73	73.6%	60.3%	0.61 (0.43–0.86)	0.005
No Parametrial Invasion	50	52	91.2%	84.6%	0.58 (0.28–1.19)	0.13
Parametrial Invasion	70	68	85.7%	72.1%	0.47 (0.29–0.77)	0.004

HR, hazard ratio; CI, confidence interval. HRs and 95% CIs are provided from univariable Cox models within each subgroup. P for interaction was derived from a model including an interaction term between the treatment group and the subgroup variable. p for interaction between treatment group and tumor size (> 4 cm vs. ≤4 cm) for overall survival = 0.048.

### 3.4 3D-PTIB demonstrates superior efficacy in OS and LC

**Table 4** shows that the 3D-PTIB cohort achieved statistically significant improvements in 3-year overall survival (OS: 76.7% vs. 66.2%; HR = 0.69, 95% CI: 0.51–0.94; p = 0.02) and local control (LC: 90.0% vs. 78.3%; HR = 0.47, 95% CI: 0.28–0.81; p = 0.01) compared with the F-3DIB cohort. Although PFS demonstrated a nonsignificant trend toward 3D-PTIB (70.8% vs. 62.3%; HR = 0.73, 95% CI: 0.53–1.00; p = 0.05), a substantial absolute improvement of 11.7% in local control was achieved with 3D-PTIB.

**Table 4:** 3D-PTIB significantly improves overall survival and local control in LACC patients: A 3-year follow-up analysis.

Endpoint	3D-PTIB (n = 120)	F-3DIB (n = 120)	Hazard ratio (95% CI)	*p*
Overall Survival (OS)	76.7%	66.2%	0.69 (0.51–0.94)	0.02
Progression-Free Survival (PFS)	70.8%	62.3%	0.73 (0.53–1.00)	0.05
Local Control (LC)	90.0%	78.3%	0.47 (0.28–0.81)	0.01

### 3.5 Multivariable analysis of survival outcomes

**Table 5** shows that multivariable Cox regression analyses, adjusted for age, FIGO stage, tumor diameter, parametrial invasion, lymph node status, and chemotherapy completion, demonstrated that 3D-PTIB remained an independent predictor of improved overall survival (aHR = 0.71, 95% CI: 0.52–0.97, p = 0.03). Furthermore, each 5-Gy increment in HR-CTV D90 was independently associated with reduced mortality (aHR = 0.82, 95% CI: 0.74--0.91, p<0.001), confirming that the survival benefit of 3D-PTIB is attributable not only to patient selection but also to its dosimetric superiority.

**Table 5:** Multivariable cox regression analysis for 3-year overall survival.

Variable	Adjusted hazard ratio (aHR)	95% CI	*P*
Treatment Modality (3D-PTIB vs. F-3DIB)	0.71	0.52 – 0.97	0.03
HR-CTV D90 (per 5 Gy increase)	0.82	0.74 – 0.91	<0.001

The model included all variables listed. Analysis based on the matched cohort (n = 240).

### 3.6 Analysis of survival outcomes by achieved HR-CTV D90 dose groups

To empirically evaluate the dose–response plateau identified by the RCS model, the matched cohort was stratified by total EQD2 of HR-CTV D90 into <85 Gy (n = 52), 85–90 Gy (n = 142), and > 90 Gy (n = 46) groups. The 3-year OS rates were 58.5%, 78.9%, and 79.3% (P<0.001), with a significant benefit for 85–90 Gy versus <85 Gy (HR = 0.48, 95% CI: 0.32–0.72, P<0.001) but not between 85–90 Gy and > 90 Gy (HR = 1.02, 95% CI: 0.62–1.67, P = 0.94). Similarly, the 3-year LC rates were 73.1%, 92.3%, and 91.3% (P<0.001), showing significant improvement for 85–90 Gy over <85 Gy (HR = 0.30, 95% CI: 0.16–0.56, P<0.001) but no further gain with > 90 Gy (HR = 1.15, 95% CI: 0.50–2.66, P = 0.74).

### 3.7 Dose–response relationship using restricted cubic splines

The dose–response relationship between the total EQD2 of HR-CTV D90 and the risk of death was further explored using adjusted RCS Cox models. The analysis revealed a nonlinear relationship (p for nonlinearity = 0.02). The hazard ratio decreased steadily with increasing D90 up to approximately 85 Gy, beyond which the curve plateaued, indicating diminishing survival returns with further dose escalation. Quantitatively, each 5-Gy increase in D90 within the steep part of the curve (below 85 Gy) was associated with an 18% reduction in mortality risk (HR = 0.82, 95% CI: 0.74–0.91, p < 0.001), consistent with the findings from the stratified dose-group analysis.

## 4 Discussion and conclusion

This single-center retrospective study highlights the significant benefits of 3D-printed template-guided interstitial brachytherapy over conventional freehand interstitial brachytherapy in patients with LACC. 3D-printed template-guided interstitial brachytherapy demonstrated a 9.2% improvement in 3-year OS (76.7% vs. 66.2%, p = 0.02) and an 11.7% increase in LC (90.0% vs. 78.3%, p = 0.01). With a median follow-up of 36.2 months, our study provides robust evidence for intermediate- to long-term survival benefits. The survival benefit is likely driven by two mechanisms (1): precise dose escalation to the HR-CTV and (2) systematic reduction in OAR exposure, underscoring the clinical value of 3D-printed template-guided interstitial brachytherapy in improving outcomes for LACC patients.

The mean HR-CTV D90 of 87.5 Gy in the 3D-PTIB group not only met but also exceeded the GEC-ESTRO recommended threshold of ≥ 85 Gy, achieving a significant 5.4 Gy advantage over the 82.1 Gy delivered with conventional freehand interstitial brachytherapy (p<0.001). This dosimetric advantage proved to be an independent driver of survival. Our multivariable analysis, which controlled for tumor size, FIGO stage, chemotherapy compliance, and other key confounders, revealed that each 5 Gy increase in D90 was associated with a significant 17% reduction in mortality risk (aHR = 0.83, 95% CI: 0.75–0.92, p<0.001). This 5.4 Gy increase represents a critical therapeutic gain, as dose–response analyses revealed that each 5 Gy increase in D90 reduces mortality risk by 18% (HR = 0.82, 95% CI: 0.74–0.91). Notably, the restricted cubic spline analysis indicated that the dose–response curve for survival plateaued above approximately 85 Gy. This observation is corroborated by our stratified analysis, which showed no significant improvement in 3-year OS (79.3% vs. 78.9%, p = 0.94) or LC (91.3% vs. 92.3%, p = 0.74) for patients receiving > 90 Gy compared to those in the 85–90 Gy range. This provides empirical evidence for diminishing returns at higher doses, consistent with previous radiobiological models of cervical cancer and the dose saturation effect reported in series such as EMBRACE, where increases beyond optimal thresholds yielded minimal additional gain (17, 18). This underscores the importance of achieving optimal dosing while avoiding unnecessary increases that may not yield additional clinical benefit. The survival advantage of 3D-printed template-guided interstitial brachytherapy was most evident in tumors > 4 cm (HR = 0.61, p = 0.005), where conventional brachytherapy often faces challenges in achieving adequate coverage owing to irregular geometries and tumor hypoxia (19). Furthermore, from a radiobiological perspective, larger tumor volumes are associated with an increased prevalence of hypoxic regions, which confer radiosistance and necessitate higher doses for effective sterilization. While our study did not employ specific imaging biomarkers to directly map and target hypoxic subvolumes, the significant dose escalation achieved with 3D-PTIB (mean D90: 87.5 Gy vs. 82.1 Gy) and the consequent improvement in local control for tumors > 4 cm are consistent with the principle of overcoming such hypoxia-related resistance through superior dose coverage. In contrast, a smaller diameter (≤ 4 cm) did not significantly improve OS (HR = 0.89, p = 0.42) or LC (HR = 0.65, p = 0.32). Critically, the analysis by tumor size confirms that the dosimetric advantages of 3D-printed template-guided interstitial brachytherapy translate into an improved therapeutic ratio specifically for bulky tumors (> 4 cm). In this high-risk subgroup, 3D-PTIB not only provided a significant 14.2% absolute improvement in LC (85.4% vs. 71.2%, p = 0.003) but also concurrently reduced the risk of severe rectal toxicity by more than two-thirds (≥ Grade 3 proctitis: 5.6% vs. 16.4%, p = 0.03). This finding supports the recommendation that standard intracavitary brachytherapy is sufficient for limited-stage disease, reserving advanced techniques such as 3D-printed template-guided interstitial brachytherapy for patients with advanced or high-risk disease who are more likely to benefit from its precision and enhanced dosimetric control (20).

When interpreting the D90 threshold in the context of the EMBRACE-I study, important technical distinctions between the trials should be considered. EMBRACE-I utilized MRI-guided adaptive brachytherapy with intracavitary ± interstitial applicators, enabling serial target volume adaptation based on superior soft-tissue visualization. In contrast, our study employed CT-based planning and 3D-printed interstitial templates. Although CT may offer less precise delineation of parametrial extension compared to MRI, potentially affecting the absolute volume and boundaries of the HR-CTV, our 3D-printed template-guided interstitial brachytherapy protocol achieved a mean D90 (87.5 Gy) comparable to the EMBRACE-I benchmark. This suggests that the meticulous preplanning and reproducible needle placement afforded by 3D-printed templates can compensate, in part, for the absence of real-time MRI adaptation in resource-constrained settings. Furthermore, the EBRT techniques in both studies (IMRT in ours, conformal techniques in EMBRACE-I) represent contemporary standards, aiming for homogeneous dose delivery prior to brachytherapy. Therefore, while the direct numerical comparability of D90 values between studies with different imaging foundations requires caution, the concordance in the direction and magnitude of the dose–response effect strengthens the biological plausibility of our findings and underscores the clinical utility of 3D-printed template-guided interstitial brachytherapy as a viable strategy to escalate dose within the framework established by high-quality evidence.

The observed survival benefit with 3D-printed template-guided interstitial brachytherapy was achieved without increasing OAR doses; conversely, significant reductions in rectal, bladder, and sigmoid D2 cc were documented (**Table 2**). This apparent paradox—simultaneous escalation in target dose and reduction in OAR exposure—is attributable to the precision inherent in 3D−printed template guidance. By pre−planning needle trajectories based on patient−specific anatomy, 3D−PTIB enables optimized spatial distribution of dwell positions, concentrating dose within the HR−CTV while avoiding OARs. Our data align with dosimetric studies of 3D−printed templates in cervical brachytherapy, which consistently report improved conformity indices and reduced OAR doses compared to freehand or standardized template approaches.

3D-printed template-guided interstitial brachytherapy significantly reduced the incidence of rectal D2 cc by 7.8 Gy (64.5 Gy vs. 72.3 Gy, p<0.001), resulting in a 60% reduction in the incidence of grade ≥ 3 radiation (4.2% vs. 11.7%, p = 0.008). This aligns with established dose-volume relationships for rectal toxicity, where maintaining D2 cc below thresholds such as 65–75 Gy EQD2 is associated with lower risks of severe complications. Similarly, the lower bladder D2 cc achieved with 3D-PTIB (78.2 Gy vs. 85.6 Gy, p<0.001) likely contributed to a marked reduction in vesicovaginal fistula rates (0.8% vs. 3.3%, p = 0.03), a severe complication that significantly impairs quality of life (21). A follow-up analysis of rectal toxicity revealed that the 3-year fistula risk was 12.5% with D2 cc ≥ 75 Gy, and patients had a twofold lower risk of proctitis with D2 cc <65 Gy (22). These outcomes highlight the critical role of 3D-printed template-guided interstitial brachytherapy in minimizing radiation-induced toxicity and improving patient outcomes. Compared with freehand techniques, 3D-printed template-guided interstitial brachytherapy has clear advantages, with a 3-year OS rate of 76.7% compared with 66.2% (p = 0.02). Additionally, the LC rate of 90.0% achieved with 3D-printed template-guided interstitial brachytherapy surpasses historical benchmarks of 75–80% with conventional freehand interstitial brachytherapy, nearing the 90% threshold reported in MRI-guided adaptive brachytherapy series (23). This finding suggests that 3D-printed template-guided interstitial brachytherapy may partially compensate for the lack of real-time MRI adaptation, which remains inaccessible in many regions. In addition to survival and LC, 3D-printed template-guided interstitial brachytherapy significantly reduces procedural variability, a key limitation of freehand techniques (24). A meta-analysis (25) demonstrated that freehand methods exhibit interoperator differences in needle placement of up to 12 mm, leading to unpredictable cold spots.

The differential survival benefit observed in patients with a diameter > 4 cm (HR = 0.61), supported by a significant interaction test (p for interaction = 0.048), highlights the critical role of patient selection in maximizing the advantages of 3D-printed template-guided interstitial brachytherapy. Bulk tumors often exhibit hypoxia-driven radioresistance due to HIF-1α overexpression, which requires high radiation doses for effective sterilization (26). However, conventional brachytherapy faces significant challenges in escalating doses without exceeding OAR constraints (27), a limitation effectively addressed by 3D-printed template-guided interstitial brachytherapy’s spatially optimized dose distribution. Similarly, the improved LC in patients with parametrial invasion (85.7% vs. 72.1%, p = 0.004) underscores the ability of 3D-printed template-guided interstitial brachytherapy to adequately cover lateral disease extensions, which are a common source of treatment failure (28). The 3D-printed template-guided template designs specifically prioritized lateral needle channels, ensuring comprehensive coverage of the cardinal ligaments, which demonstrated the importance of tailored applicator designs for addressing parametrial disease. These findings collectively illustrate how 3D-printed template-guided interstitial brachytherapy overcomes key challenges in brachytherapy, offering superior outcomes for high-risk patient subgroups. Despite its demonstrated advantages, 3D-printed template-guided interstitial brachytherapy remains underutilized in low-resource settings, particularly in regions with high cervical cancer mortality.

Additionally, our study included patients with challenging posterior tumor−rectum relationships, which are often associated with higher risks of underdosage or rectal toxicity. Through the use of angulated needle trajectories, stricter rectal wall dose constraints, and intraoperative imaging verification, we were able to achieve adequate posterior coverage without increasing rectal complications, as reflected in the low incidence of ≥ Grade 3 proctitis (4.2%) and rectal fistula (0.8%) in the 3D−PTIB group. This suggests that 3D−PTIB, when combined with meticulous pre−planning and real−time imaging, can safely address high−risk anatomical scenarios that are typically problematic in freehand approaches.

The intentional focal undosage combined with EBRT boost represents a pragmatic and patient-specific solution for a small subset of anatomically extreme LACC cases (5.3% in our series). This strategy acknowledges the physical and biological limits of brachytherapy when tumor proximity to OARs is intimate. By accepting a controlled, well-defined underdosage in the brachytherapy plan and compensating with a highly conformal external beam boost, we aimed to approximate the therapeutic dose while rigorously protecting OARs. This approach aligns with the ALARA (As Low As Reasonably Achievable) principle for normal tissues and is supported by studies demonstrating that moderate dose compromises in small volumes may be offset by the spatial fractionation advantage of EBRT. However, it must be emphasized that this is a compromise; the radiobiological superiority of brachytherapy’s high dose rate and steep dose gradient is partially forfeited. The long-term outcomes for these patients (3-year OS: 62.5% in this small subgroup) suggest that while the strategy may avert severe toxicity, it may not fully recapture the survival advantage seen in patients where full-dose 3D-printed template-guided interstitial brachytherapy was feasible. This underscores the critical importance of patient selection and the primary goal of maximizing brachytherapy coverage whenever anatomically possible.

Based on dosimetric and survival evidence alongside established brachytherapy principles, 3D-printed template-guided interstitial brachytherapy should be prioritized over standard ICBT in locally advanced cervical cancer patients meeting any objective criterion: primary tumor diameter > 4 cm or post-EBRT HR-CTV > 30 cm³; inability of ICBT on virtual planning to achieve HR-CTV D90 ≥ 85 Gy EQD2 within standard OAR limits (rectum D2 cc <70–75 Gy, bladder D2 cc <90 Gy); extensive or asymmetric parametrial invasion requiring customized dose distribution; or tumor involvement of the lower vaginal third/significant vaginal stenosis compromising intracavitary applicator placement. For tumors ≤ 4 cm with symmetric morphology and no parametrial extension, ICBT remains effective.

This retrospective study has inherent limitations, including potential residual confounding despite PSM and the possibility of selection bias. The preferential allocation of more complex tumors to the 3D-printed template-guided technique, designed for such cases, may have biased results toward the null. The observed significant survival benefit for 3D-printed template-guided interstitial brachytherapy despite this potential bias supports the robustness of the finding, as does the consistent dose–response relationship. Second, although we addressed pubic arch interference and sacrum/iliac wing interference through preoperative simulation and template optimization as described, extreme anatomical variations or tumor locations that precluded the identification of any safe, dosimetrically adequate needle path were considered contraindications for standalone 3D-printed template-guided interstitial brachytherapy in our protocol. These patients were managed with hybrid techniques or EBRT boost and excluded from the analysis, which ensures that the reported outcomes reflect the efficacy of 3D-printed template-guided interstitial brachytherapy when technically feasible but may limit the generalizability of our findings to all-comers with LACC. Third, while the median follow-up of 3 years is adequate to assess 3-year survival and most relevant late toxicities, it is relatively short for evaluating very late toxicities, such as secondary malignancies, which account for 5-10% of deaths beyond 10 years. While the primary outcome (OS) was prespecified, the evaluation of multiple secondary endpoints (PFS, LC, various toxicities) and exploratory subgroup analyses increased the risk of type I error due to multiple comparisons. Although we reported unadjusted p values for transparency, findings for secondary outcomes (particularly the borderline PFS result, p = 0.05) and the subgroup analyses presented in **Table 3** should be interpreted as exploratory and hypothesis-generating. Fourth, the reliance on CT for final planning and target delineation in the F-3DIB group versus the use of MRI-based preplanning in the 3D-PTIB group introduces fundamental imaging heterogeneity. As acknowledged, CT may underestimate HR-CTV volumes, especially in parametrial regions, compared to MRI. This could lead to a systematic bias where the F-3DIB group’s target volumes might be smaller or less accurately defined than the biological true volume. Fifth, our toxicity assessment, while reporting valuable incidence data, did not capture the time-to-event for each severe toxicity. The follow-up data collection window was designed to ensure adequate assessment of 3-year survival endpoints, and the specific dates of onset for all ≥ Grade 3 toxicities were not systematically recorded. Therefore, we were unable to generate Kaplan–Meier curves or report median times to toxicity occurrence, which represents a limitation in fully characterizing the temporal profile of late adverse events. Future prospective studies with dedicated toxicity follow-up protocols will address this aspect.

While retrospective data suggest a dose–response plateau for HR-CTV D90 beyond 85 Gy, this requires prospective validation. We propose a multicenter trial combining the reproducibility of 3D-printed templates with the adaptive precision of MRI-guided brachytherapy. The protocol mandates MRI-based planning at each fraction for adaptive delineation of targets and organs at risk. A dose-adaptive randomized design will compare a standardized arm against a response-adapted arm, permitting escalation above 90 Gy only if OAR constraints are met or resistant subvolumes are identified via functional imaging. This will evaluate the efficacy plateau and assess whether the integrated approach can safely extend the therapeutic index. The generalizability of the current findings is limited by their single-center, retrospective nature; external validation in diverse settings is essential. Future prospective, multicenter trials with comparative designs are needed to establish causal efficacy and optimize patient selection.

This study positions 3D-printed template-guided interstitial brachytherapy as a modality for locally advanced cervical cancer, especially in high-risk subgroups with bulky tumors or parametrial invasion. By combining precision dosimetry with operational feasibility, a scalable solution to improve outcomes in diverse healthcare settings can be developed.

## Data Availability

The datasets presented in this study can be found in online repositories. The names of the repository/repositories and accession number(s) can be found below: The raw data are available at https://www.jianguoyun.com/p/DSgYuqEQuaiFChiQkZMGIAA.

## Glossary

3D-PTIB: Three-dimensional printed template-guided interstitial brachytherapy
F-3DIB: Conventional freehand interstitial brachytherapy
LACC: Locally advanced cervical cancer
FIGO: International Federation of Gynecology and Obstetrics
OS: Overall survival
PFS: Progression-free survival
LC: Local control
HR: Hazard ratio
HR-CTV: High-risk clinical target volume
IR-CTV: Intermediate-risk clinical target volume
OAR: Organ at risk
D90: Dose covering 90% of HR-CTV
D2 cc: Minimum dose delivered to the most exposed 2 cc of an OAR
V100: Volume receiving 100% of the prescribed dose
CI: Conformity index
CCRT: Concurrent chemoradiation therapy
ICBT: Intracavitary brachytherapy
ISBT: Interstitial brachytherapy
IMRT: Intracavitary-modulated radiotherapy
EBRT: External beam radiotherapy
GEC: Groupe Européen de Curiethérapie–European Society for Therapeutic Radiology and Oncology
